# Diversity of Orchid Bees in Mangroves Under Anthropogenic Pressure: A Study in Bay of Panamá and Bay of Chame

**DOI:** 10.3390/insects17010085

**Published:** 2026-01-13

**Authors:** Jeancarlos Abrego, Anette Garrido-Trujillo, José A. Rivera, Alonso Santos Murgas

**Affiliations:** 1 Departamento de Zoología, Escuela de Biología, Facultad de Ciencias Naturales, Exactas y Tecnología, Universidad de Panamá, Ciudad de Panamá 3366, Panama; jean.abrego-l@up.ac.pa (J.A.); jriveralorenzo@gmail.com (J.A.R.); 2 Departamento de Genética y Biología Molecular, Escuela de Biología, Facultad de Ciencias Naturales, Exactas y Tecnología, Universidad de Panamá, Ciudad de Panamá 3366, Panama; anecgarrido@gmail.com; 3 Estación Científica Coiba AIP, Ciudad del Saber 3366, Panama

**Keywords:** Euglossini bees, conservation, deforestation, fragmentation, mangrove forests, perturbation

## Abstract

Mangrove ecosystems along the Pacific coast of Panama are increasingly exposed to anthropogenic pressures such as urban expansion and deforestation. These habitats provide resources for orchid bees (tribe Euglossini), yet information on their assemblages in mangrove environments remains limited. In this study, we documented the diversity and composition of orchid bee communities in mangrove–forest edges from two coastal areas with contrasting levels of human disturbance: Panama Bay and Chame Bay. Orchid bee sampling was carried out during two independent periods: from April to July 2022 at three sites in Panama Bay, and from December 2022 to January 2023 at one site in Panama Bay and one site in Chame Bay, using McPhail traps baited with eucalyptus oil and distributed across multiple zones within each site. A total of 427 individuals representing 14 species and three genera were recorded. Observed species richness and abundance were lower at the more urbanized mangrove sites, where collections were dominated by a few widespread species, particularly *Eulaema nigrita*. Multivariate analyses revealed differences in community composition between sites. These patterns suggest associations between anthropogenic context and orchid bee assemblage structure in mangrove edges, although longer-term and multi-method studies are required to evaluate temporal consistency and underlying mechanisms. This study provides baseline information to support future monitoring of orchid bee communities in tropical coastal ecosystems.

## Simple Summary

Mangrove forests in Panama support a wide range of organisms, including orchid bees, which are important pollinators in tropical ecosystems. However, many mangrove areas are increasingly exposed to human activities such as urban development, deforestation, and pollution. In this study, we compared orchid bee assemblages in two mangrove systems with contrasting levels of human disturbance: one located near Panama City and another in a less urbanized coastal area. Sampling of orchid bees was conducted during two independent periods between 2022 and 2023, covering different localities in Panama Bay and Chame Bay, using scent-baited traps, and differences in species richness, abundance, and community composition were documented. The more urbanized mangrove edge showed lower observed richness and a community dominated by a few widespread species, whereas the less disturbed site exhibited a more even assemblage. These results describe patterns in orchid bee communities under different anthropogenic contexts and provide baseline information to support future long-term monitoring and conservation efforts in tropical coastal mangroves.

## 1. Introduction

Orchid bees (Euglossini, Apidae: Hymenoptera) are brightly colored pollinators endemic to the Neotropics [1]. Known for their iridescent hues and perfume-collecting behavior, these bees play a vital role in pollinating a wide range of tropical plant species, especially orchids [2]. Although the tribe includes five genera, only four occur naturally in Mesoamerica (*Euglossa* Latreille, 1802, *Eulaema* Lepeletier, 1841, *Eufriesea* Cockerell, 1908, *Exaerete* Hoffmannsegg, 1817); and one is only known for South America (*Aglae* Lepeletier & Serville, 1828). Approximately 250 described species are distributed from sea level to elevations above 2000 m [2,3]. Unlike other corbiculate bees, orchid bees are solitary and exhibit distinct nesting and foraging behaviors [4]. Females of *Euglossa* species possess a corbicula on the hind tibia, while males show a thickening in the same area. Although they do not form colonies, some species may establish communal nests in natural cavities, including abandoned nests of other bees [5].

Mangroves are intertidal, halophytic forests dominated in Panama by *Rhizophora mangle* Linnaeus, 1753, *Avicennia germinans* Linnaeus, 1764*, Laguncularia racemosa* (L.) C. F. Gaertn., 1807, and *Conocarpus erectus* Linnaeus, 1753, arranged along seaward-to-landward gradients that differ in salinity, inundation frequency, canopy structure, and proximity to adjacent vegetation. Such gradients and their adjacency to larger forest matrices may influence local orchid bee assemblages, even if foraging ranges extend well beyond mangrove habitats [6]. Within mangrove areas, other plant species can also be found, such as *Brassavola nodosa* (L.) Lindl, 1831, as well as ferns and bromeliads [7]. Although a comprehensive inventory of all plant species composing the mangrove vegetation is not yet available, palynological studies have reported the use by orchid bees of up to 43 genera belonging to 23 plant families, including mangrove taxa such as those mentioned above [8].

Panama and Costa Rica are considered hotspots of Euglossini diversity, together hosting over 70 species reported for Central America and southern Mexico [2,9]. In Panama, orchid bees have been highlighted not only as key pollinators but also as bioindicators of forest health due to their sensitivity to habitat disturbance [10,11]. However, rapid urban expansion, deforestation, and pollution along Panama’s Pacific coast—particularly around Panama Bay—have significantly altered mangrove ecosystems [12,13]. These changes may reduce the availability of floral and nesting resources, ultimately impacting the composition and abundance of orchid bee communities [14,15].

Despite their ecological importance, there is a lack of research focused specifically on Euglossini communities in mangrove habitats of Panama. Given their role as sensitive indicators of environmental change, studying their response to varying degrees of anthropogenic disturbance is crucial for developing informed conservation strategies [10,16]. This study aims to compare the composition and diversity of orchid bee communities in two mangrove systems under contrasting levels of human disturbance: Panama Bay (urbanized) and Chame Bay (conserved). We hypothesize that the urbanized site will present lower species richness and evenness, with a community structure dominated by generalist or disturbance-tolerant species. Long-term records from central Panama show seasonal fluctuations but overall resilient orchid bee populations, even across El Niño Southern Oscillation (ENSO) cycles, with stable distribution of abundances over decades. We therefore frame our results as a short-term baseline within this broader context [17,18,19,20].

## 2. Materials and Methods

### 2.1. Study Area

Panama Bay, located south of the province of the same name, is a vast body of water forming part of the Gulf of Panama. With an approximate length of 96 km and a maximum width of 35 km, its coasts include Panama City and the Pacific entrance to the Panama Canal. This ecosystem comprises estuaries, mangroves, floodplain forests, marshes, and freshwater lagoons [21]. It is recognized as a site of international importance by Ramsar Convention since 2003 [22], serving as a habitat for one to two million migratory shorebirds, including species such as the western sandpiper (*Calidris mauri* Cabanis, 1857) and the semipalmated plover (*Charadrius semipalmatus* Bonaparte, 1825) [23]. However, the bay faces significant environmental challenges due to urban development, pollution, and mangrove deforestation, leading to alterations in its ecological characteristics [12].

On the other hand, Chame Bay is located on the Pacific slope of Panama and is a semi-enclosed body of water with a system of shallow channels. It receives inputs from rivers and streams with low sedimentation, which has allowed the development of one of the best-preserved mangrove ecosystems on Panama’s Pacific coast [21]. This mangrove forest extends from the mouth of the Chame River to the Monte Oscuro district, covering more than 8000 hectares [13]. Unlike Panama Bay, Chame Bay has experienced less human intervention, contributing to the preservation of its biodiversity and ecological functions. Nevertheless, recent studies have emphasized the need to monitor and manage human activities in the region to ensure the conservation of this valuable ecosystem [21].

**Figure 1:**
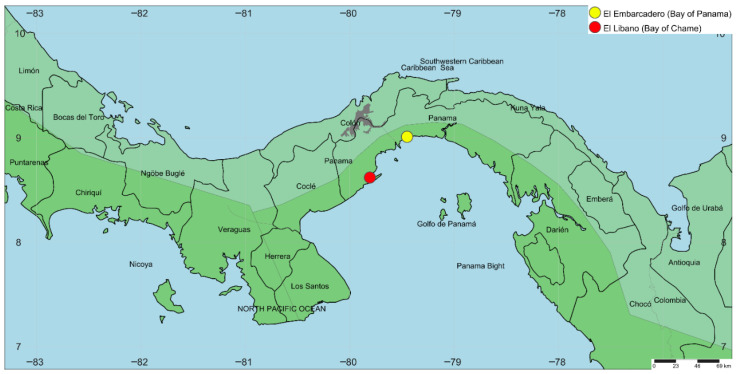
Map of the sampling area of orchid bees in Panama Bay and Chame Bay.

**Figure 2:**
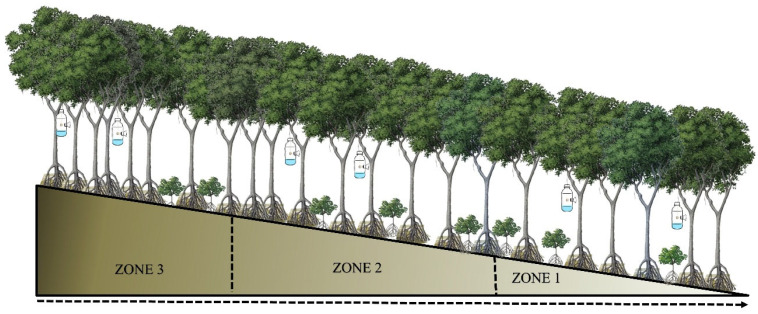
Arrangement of the sampling zones inside the mangrove forest of Panama Bay and Chame Bay. Zone 1 is closest to the shoreline, zone 2 intermediate area, and zone 3 the closest to the tropical forest near the mangroves.

The samples were collected in four areas located in Panama Bay and Chame Bay: El Embarcadero, Don Bosco and Costa Sur, situated in the Panama Bay (coordinates 9.019036, −79.436975); and El Líbano, located in the Bay of Chame (coordinates 8.620592, −79.807068) (see Figure 1). In all locations, a plot was placed measuring 0.4 km^2^ (200 m long by 200 m wide). Each plot extended from the tidal region to the mainland, and was divided into three zones: Zone 1, was the closest to the shoreline; Zone 2, was positioned midway between the tidal area and the forest; and Zone 3, was the transition zone between the mangrove and the forest (Figure 2).

### 2.2. Sampling of Orchid Bees (Euglossini)

Sampling was conducted during two independent time frames. The first sampling period took place from April to July 2022 exclusively in Panama Bay (El Embarcadero, Don Bosco, and Costa Sur). The second sampling period occurred from December 2022 to January 2023 at El Embarcadero (Panama Bay) and El Líbano (Chame Bay). We acknowledge that a six-month window cannot capture full annual turnover; our design provides a baseline snapshot that should be complemented by year-round and multi-year monitoring. It is also worth noting that sampling at El Líbano was attempted for a longer time frame, but on two occasions the traps were destroyed due to habitat disturbance: the mangrove forest had been cut down for charcoal production. Two McPhail traps were installed per zone, totaling six traps per plot, with identical spatial distribution and trap height (~1.5 m above ground). Samples were collected every two weeks, resulting in a total of 12 collection events per trap at each plot. This standardized sampling effort ensured comparability between sites in terms of trapping intensity, duration, and spatial coverage. Traps were baited exclusively with eucalyptus oil and remained continuously active throughout the study period. Pharmaceutical eucalyptus oil was used (eucalyptol mixture rather than pure 1,8-cineole), using the same product across sites for methodological consistency. We note that using pure 1,8-cineole and sampling the canopy can broaden species detection; both choices represent deliberate limitations we now acknowledge and recommend for future work. The samples were then taken to the Museo de Invertebrados G. B. Fairchild at the Universidad de Panamá (MIUP) for processing. Specimens were mounted on No. 2 pins, labeled, and identified using the taxonomic key in [2]. The specimens analyzed in this study were collected under collection permit ARB-133-2022, granted by the corresponding environmental authorities. All orchid bee specimens were preserved and deposited as voucher specimens in the entomological collection of the Museo de Invertebrados G. B. Fairchild at the Univesidad de Panamá (MIUP-HEU). The deposited vouchers are cataloged under the reference numbers MIUP-HEU-0001 to MIUP-HEU-0427, ensuring proper documentation and availability for future taxonomic and ecological studies. All specimens were preserved under controlled humidity and temperature conditions, following the curation protocols of the MIUP collection.

### 2.3. Data Analysis

All statistical analyses were conducted using R software (version 4.3.1) [24]. Alpha diversity metrics—species richness, Shannon index, Simpson index, Fisher’s alpha, and Pielou’s evenness—were calculated using the vegan and BiodiversityR packages (version 2.17.4) to assess species heterogeneity within sites. To estimate and compare species richness relative to sampling effort, species accumulation and rarefaction curves were generated using the specaccum and rarecurve functions from the vegan package (version 2.7.2). Beta diversity was evaluated using the Bray–Curtis dissimilarity and Jaccard similarity index, calculated with the vegdist function in vegan, to assess species turnover and community differentiation between sites. These dissimilarity matrices were used to perform a Principal Coordinates Analysis (PCoA) with the cmdscale function, and visualizations were generated using ggplot2 package (version 4.0.1).

To assess the effect of site on total species abundance, a Generalized Linear Mixed Model (GLMM) was fitted using the glmmTMB package (version 1.1.3). The model assumed a negative binomial distribution to account for overdispersion of orchid bees, with site as a fixed effect and sampling zone as a random effect. The significance of the model was assessed using Wald chi-square tests via the Anova function from the car package (version 3.1.3). All plots were created using the ggplot2 and ggpubr packages (version 0.6.2).

## 3. Results

### 3.1. General Samples

**Figure 3:**
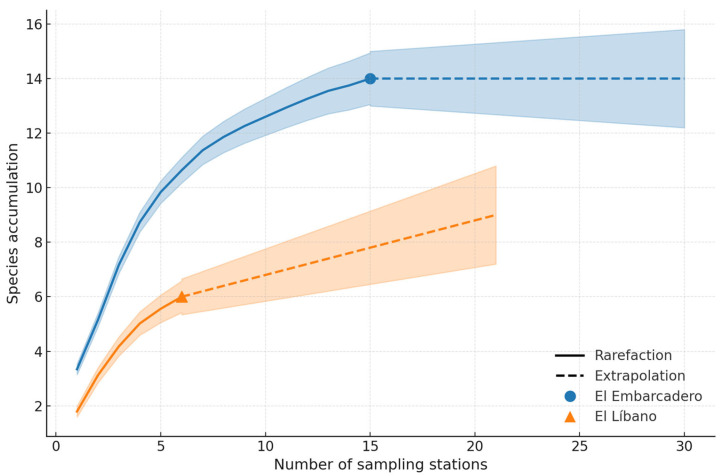
Species accumulation and rarefaction curves in El Embarcadero and El Líbano; the species accumulation curve shows the total number of recorded species as the number of samples increases, with a tendency to stabilize, indicating that the sampling has captured a substantial portion of the expected diversity. The rarefaction curve represents the estimated species richness as a function of sampling effort, allowing for a more equitable comparison of diversity between sites and highlighting differences in the number of detected species.

Across the two sampling periods conducted between 2022 and 2023 in Panama Bay and Chame Bay, a total of 427 orchid bee specimens were collected, belonging to 14 species in three genera (*Euglossa*, *Exaerete*, and *Eulaema*). Of these, nine species were recorded across multiple sites, with *Eulaema nigrita* being the most abundant species overall (246 individuals). The genus *Euglossa* presented 83 individuals and 12 species, the genus *Eulaema* presented 219 individuals and one species, and the genus *Exaerete* presented one species and 17 individuals (see Table 1). The species accumulation and rarefaction curves reveal key patterns in species richness at El Embarcadero and El Líbano. The species accumulation curve indicates that as the number of samples increases, the accumulated number of species also grows, although it tends to stabilize, suggesting that the sampling captured a substantial portion of the detectable diversity under our design, but not necessarily the full expected richness of the assemblage. On the other hand, the rarefaction curve shows the estimated species richness as a function of sampling effort, highlighting differences in diversity between sites and allowing for a more equitable comparison of the number of detected species (Figure 3). In El Líbano, Bahía de Chame, a total of 59 individuals from three genera (*Euglossa*, *Exaerete*, *Eulaema*) were collected, distributed across six species (*Euglossa deceptrix*, *Euglossa imperialis*, *Euglossa variabilis*, *Eulaema meriana*, *Eulaema nigrita*, and *Exaerete smaragdina*).

### 3.2. Panama Bay (Don Bosco, El Embarcadero, Costa Sur)

In Panama Bay, sampling was conducted at three sites; El Embarcadero, Don Bosco, and Costa Sur, during April, May, June, and July 2022, yielding a total of 319 orchid bee individuals. El Embarcadero showed the highest abundance, with 226 individuals, whereas Costa Sur and Don Bosco exhibited lower abundances, with 70 and 23 individuals, respectively. These reduced abundances at the latter sites may indicate limited habitat capacity to support diverse and stable orchid bee assemblages, potentially affecting long-term population viability. Species richness varied among sites, with the highest diversity observed at El Embarcadero (nine species). In contrast, Costa Sur and Don Bosco presented lower richness, with six and four species, respectively (Table 2). Across the three sites, the distribution of individuals among sampling zones showed notable variation. Overall, Zone 2 recorded the highest abundance (124 individuals), followed by Zone 3 (107 individuals) and Zone 1 (88 individuals). However, this pattern was not consistent across all sites. In Costa Sur, Zone 2 had the highest abundance, whereas in Don Bosco, Zone 1 recorded the greatest number of individuals. In El Embarcadero, Zone 3 exhibited the highest abundance. Based on the combined Panama Bay data, a total of 10 species were recorded. Five species were restricted to a single site: *Euglossa cognata* occurred only in Costa Sur, while *Euglossa allosticta*, *Euglossa disimula*, *Euglossa hemichlora*, and *Euglossa tridentata* were recorded exclusively in El Embarcadero. The remaining five species (*Euglossa cybelia*, *Euglossa dressleri*, *Euglossa imperialis*, *Eulaema nigrita*, and *Exaerete smaragdina*) were detected in more than one site, indicating a balanced representation of site-specific and more widely distributed species within Panama Bay (Table 1).

Three genera were collected: *Euglossa* Latreille, 1802; *Exaerete* Hoffmannsegg, 1817; and *Eulaema* Lepeletier, 1841; *Eulaema* being the most abundant genus with 219 individuals, representing 68.6% of the total sampling. The genus *Euglossa* accounted for a total of 83 individuals, and *Exaerete* for a total of 17 individuals. The genus with the highest species richness was *Euglossa* with ten species, while *Eulaema* and *Exaerete* each had one species (Table 2).

### 3.3. Comparison Between El Líbano and El Embarcadero During the Same Period (Two Months)

**Figure 4:**
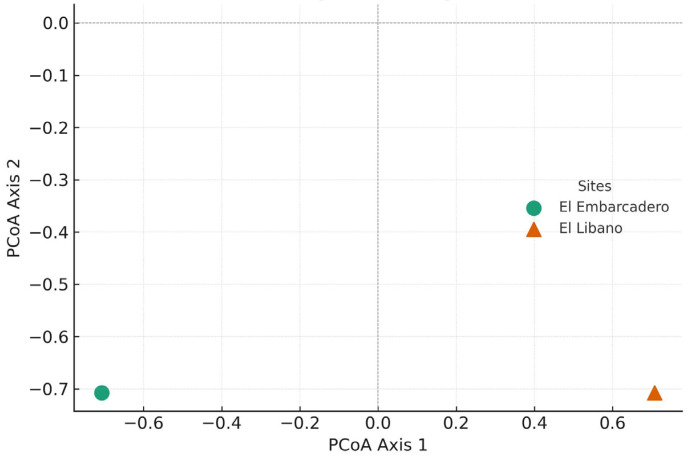
PCoA based on Jaccard visualizes compositional differences.

When comparing only the overlapping two-month period (December 2022 to January 2023), El Líbano yielded 59 individuals and El Embarcadero 49, for a total of 108 individuals. Of these, 75 belonged to the genus *Euglossa*, 32 to *Eulaema*, and one to *Exaerete*. Overall richness was 12 species distributed across three genera, with six species recorded in El Líbano and nine in El Embarcadero. Regarding genus distribution, nine species of *Euglossa*, two of *Eulaema*, and one of *Exaerete* were identified (Table 3). The PCoA analysis illustrates that El Embarcadero (circle) and El Líbano (cross) are separated in the bidimensional space, suggesting compositional differences between the two sites (Figure 4).

**Figure 5:**
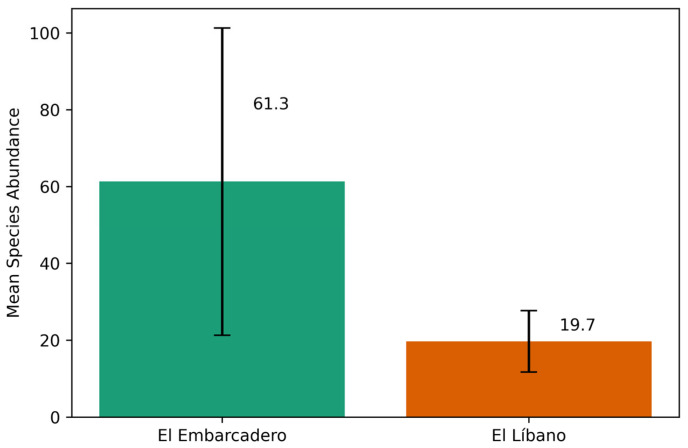
Mean species abundance (±SE) in two mangrove sampling sites in Panama: El Embarcadero (Bay of Panama) and El Líbano (Bay of Chame). Bars represent the mean number of individuals per sampling event. A generalized linear mixed model (GLMM) showed that El Líbano had significantly lower species abundance compared to El Embarcadero (coefficient = −0.9832, *p* < 0.001), indicating a ~62% reduction in species abundance.

The GLMM analysis comparing species abundance between El Embarcadero and El Líbano shows a significant difference, with El Líbano having a lower species abundance compared to El Embarcadero (coefficient = −0.9832, *p* < 0.001). This indicates that species abundance in El Líbano is approximately 62% lower than in El Embarcadero. This discrepancy arises because the GLMM was fitted on per-event means, not on raw totals; thus, although cumulative totals were slightly higher at El Líbano, the average abundance per sampling event was significantly lower compared to El Embarcadero (Figure 5).

**Figure 6:**
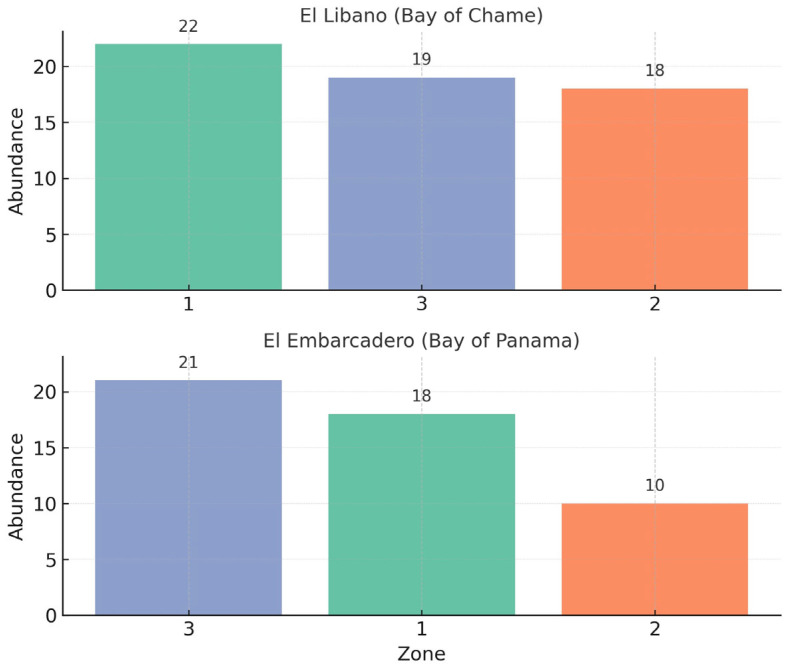
Abundance per sampling zone in the mangrove forest of Bay of Panama and Bay of Chame.

At the site of El Embarcadero, Bay of Panama, a total of nine species were collected: eight of *Euglossa* and one of *Eulaema*, with an abundance of 34 and 15 individuals, respectively. Regarding the sampling zone, the highest abundance was observed in Zone 3 with 21 individuals, followed by Zone 1 with 18, and Zone 2 with 10 individuals. In terms of species’ richness, Zone 3 had a total of seven species, while Zones 1 and 2 each had a richness of four species. For El Líbano, Bay of Chame, a total of six species were collected: three of Euglossa, one of *Eulaema*, and one of *Exaerete*, with an abundance of 41 individuals, 17 individuals, and one individual, respectively. Regarding the sampling zone, the highest abundance was recorded in Zone 1 with 22 individuals, followed by Zone 3 with 19, and finally, Zone 2 with 18 individuals (Figure 6).

Statistical analyses were performed to determine the alpha (α) and beta (β) diversity indices of the sampled zones (Table 4). The Shannon Diversity Index was found to be 1.014 for El Líbano and 1.863 for El Embarcadero. Additionally, the Bray–Curtis index was 0.7407, and the Jaccard index was 0.750 (Table 5).

Although total captures across the full sampling period were higher at El Embarcadero (319 individuals), here we restricted the comparison to the overlapping months with El Líbano to ensure equal sampling effort.

## 4. Discussion

Across the two non-continuous sampling periods, we recorded differences in orchid bee assemblages between sites and bays, as well as the presence and relative abundance of orchid bees along mangrove–forest edges exposed to different degrees of urbanization. Even though orchid bees are not restricted to mangrove habitats, we used mangrove–forest edges as a coastal, understudied context to document assemblage structure under urban pressure. Given the limited sampling period, we refrain from making causal inferences and interpret the observed site differences as preliminary patterns requiring year-round and multi-year validation. This supports the hypothesis that anthropogenic pressures—such as urban expansion, deforestation, and habitat fragmentation—may be associated with reduced diversity and shifts in assemblage structure in coastal orchid bee communities [25]. The highest richness and abundance were observed in El Embarcadero, particularly in the transition zone between mangrove and adjacent forest. In contrast, Don Bosco, the most urbanized site, exhibited the lowest diversity metrics. These patterns are consistent with previous research indicating that habitat degradation reduces the availability of floral resources and suitable nesting sites, thereby reshaping pollinator community composition [26]. Furthermore, recent evidence shows that local-scale landscape simplification can negatively affect Euglossini bee assemblages, with community descriptors declining as landscape configuration increases. This pattern suggests that increased landscape heterogeneity in urbanized coastal zones may lead to a simplification of native forest habitats, ultimately driving the loss of orchid bee biodiversity. Similar studies have also linked these changes in landscape structure to altered connectivity and reduced persistence of specialized bee taxa in disturbed environments [27]

The dominance of *Eulaema nigrita*, a generalist species known for its tolerance to disturbed environments [28,29], and the low Shannon and Simpson indices found in El Líbano (Bay of Chame) suggest a community shift toward disturbance-resilient taxa. Meanwhile, specialist species were comparatively less represented in our samples, a pattern consistent with assemblage shifts observed in disturbed landscapes, although our design does not allow identification of underlying mechanisms. Although some variation in species composition was observed among sites, overall beta diversity was modest, indicating limited heterogeneity. These results suggest that current environmental conditions may be associated with lower observed richness and evenness in orchid bee assemblages at more urbanized mangrove edges. However, longer-term and mechanistic studies are required to evaluate whether such patterns persist through time or influence population stability [30,31]. Our findings are consistent with long-term observations that document resilient populations and recurrent dominance by a few widespread species in disturbed settings. Complementary lines of evidence, such as palynology from foragers and nest records (including *Eufriesea* nesting in urban structures), help explain resource use and nesting substrates in landscapes where mangroves abut larger forest matrices.

Using a single, non-standard lure and sampling only the understory may under-represent species with different scent preferences or vertical strata; future work should include pure 1,8-cineole plus additional attractants and a canopy–understory design. A methodological limitation to consider is the exclusive use of eucalyptus oil as an attractant. While it is a widely used and effective lure for a broad range of Euglossini species, the lack of other scent options may have biased the sampling against species with more specialized chemical preferences. Future studies should incorporate multiple attractants to capture a more complete picture of orchid bee diversity [2,23]. These findings highlight potential vulnerabilities of pollinator communities in urban mangrove ecosystems, underscoring the importance of targeted conservation strategies. Multi-seasonal and multi-site monitoring is essential to better understand temporal trends and to guide biodiversity management in increasingly disturbed coastal habitats. Given the short-term nature of our sampling and the use of a single attractant, these patterns should be interpreted as descriptive rather than mechanistic, providing a baseline for future year-round and multi-method monitoring.

## 5. Conclusions

This baseline assessment suggests associations between urban pressure and patterns of reduced richness and shifts in assemblage structure, often favoring disturbance-tolerant taxa. Establishing causality will require year-round, multi-attractant, and canopy-to-understory sampling that captures temporal and vertical variation not covered in our study. Conservation strategies should prioritize habitat protection and restoration, as well as the enhancement of floral resources within mangrove areas. Future studies should incorporate multi-attractant sampling, seasonal and multi-year monitoring, and broader landscape comparisons to refine our understanding of how orchid bee assemblages respond to environmental pressures.

## Figures and Tables

**Table 1:** Orchid bee (Euglossini) diversity and abundance per sampling site during the two sampling periods (2022–2023).

Site/Species	Zones			Total/Site
	1	2	3	
Costa Sur	10	43	17	70
*Euglossa cognata*		4		4
*Euglossa cybelia*	1	9		10
*Euglossa dressleri*	2	3		5
*Euglossa imperialis*	6			6
*Eulaema nigrita*		27	16	43
*Exaerete smaragdina*	1		1	2
Don Bosco	15	3	5	23
*E. cybelia*			1	1
*E. dressleri*			1	1
*Eulaema nigrita*	14	3	3	20
*Exaerete smaragdina*	1			1
El Embarcadero	81	87	107	275
*Euglossa allosticta*	1	2	2	5
*Euglossa deceptrix*		2	3	5
*Euglossa cybelia*		1		1
*Euglossa disimula*	2	3	10	15
*Euglossa dodsoni*	5			5
*Euglossa dressleri*	1	7		8
*Euglossa hemichlora*	2	5	5	12
*Euglossa heterosticta*			1	1
*Euglossa imperialis*		11	19	30
*Euglossa tridentata*			3	3
*Euglossa variabilis*		1	2	3
*Eulaema nigrita*	66	53	52	171
*Exaerete smaragdina*	4		10	14
*Exaerete tridentata*		2		2
El Líbano	22	18	19	59
*Euglossa deceptrix*			1	1
*Euglossa imperialis*	21	5	13	21
*Euglossa variabilis*			1	1
*Eulaema meriana*		1	4	5
*Eulaema nigrita*		12		12
*Exaerete smaragdina*	1			1
Total	128	151	148	427

**Table 2:** Orchid bee richness and abundance per sampling site (Costa Sur, Don Bosco, El Embarcadero and El Libano).

	Costa Sur	Don Bosco	El Embarcadero	El Líbano
Richness	6	4	9	6
Abundance	70	23	226	59

**Table 3:** Orchid bee richness for Bay of Chame and Bay of Panama.

Orchid Bee Species	El Embarcadero (Bay of Panama)	El Líbano (Bay of Chame)
*Euglossa allosticta* Moure, 1969	x	-
*Euglossa deceptrix* Moure, 1968	x	x
*Euglossa imperialis* Cockerell, 1922	-	x
*Euglossa disimula* Dressler, 1978	x	-
*Euglossa dodsoni* Moure, 1965	x	-
*Euglossa hemichlora* Cockerell, 1917	x	-
*Euglossa heterosticta* Moure, 1968	x	-
*Euglossa tridentata* Moure, 1970	x	-
*Euglossa variabilis* Friese, 1899	x	x
*Eulaema nigrita* Lepeletier, 1841	x	x
*Eulaema meriana* (Olivier, 1789)	-	x
*Exaerete smaragdina* (Guérin-Méneville, 1845)	-	x

x: present, -: absent.

**Table 4:** Alpha diversity indices (α) of El Líbano, Bay of Chame, and El Embarcadero, Bay of Panama.

Alpha Diversity Indices	El Líbano (Bay of Chame)	El Embarcadero (Bay of Panamá)
Richness	6	9
Abundance	59	49
Shannon	1.014	1.863
Gini-Simpson	0.5136	0.8038
Inverse Simpson Index	2.056	5.098
Fisher-Alpha	1.670	3.236
Berger-Parker	0.6610	0.3061
Pielou’s Evenness	0.5660	0.8478
E-evenness	0.4595	0.7157

**Table 5:** Beta diversity indices (β) of El Líbano, Bay of Chame, and El Embarcadero, Bay of Panama.

Sampling Sites	El Líbano (Bay of Chame)	Beta Diversity Indices
El Embarcadero (Bay of Panamá)	0.7407	Bray–Curtis
El Embarcadero (Bay of Panamá)	0.75	Jaccard

## Data Availability

The original data presented in the study are openly available in FigShare at https://doi.org/10.6084/m9.figshare.29896130 (accessed on 5 August 2025).
